# Single-stage delayed hybrid reconstruction after failed implant-based breast surgery using the pedicled thoracodorsal artery perforator flap: a report of three cases

**DOI:** 10.1080/23320885.2025.2603728

**Published:** 2025-12-16

**Authors:** Rushabh Shah, Krzysztof Sosnowski, Shoa Nayyer, Charles M. Malata

**Affiliations:** ^a^ Department of Plastic and Reconstructive Surgery, Addenbrooke’s Hospital, Cambridge University Hospitals NHS Foundation Trust, Cambridge, United Kingdom; ^b^ School of Clinical Medicine, University of Cambridge, Cambridge, United Kingdom

**Keywords:** Delayed breast reconstruction, implant failure, thoracodorsal artery perforator flap, expandable implant, hybrid reconstruction

## Abstract

The thoracodorsal artery perforator (TDAP) flap is established in immediate and early breast reconstruction, but its role in single-stage, hybrid (flap-plus-implant) reconstruction performed ≥12 months after failed implant-based surgery is not well described.

Three consecutive patients underwent pedicled TDAP flap reconstruction combined with a subpectoral Becker-35 expandable implant more than one year after failed implant-based reconstruction in scarred fields with poor skin quality. Pre-operative planning included CT angiography (CTA) for perforator mapping, complemented by handheld Doppler; intra-operative indocyanine green (ICG) angiography was unavailable. Mean flap size was 17 × 8 cm, pedicle 13–15 cm, mean operative time 247 min, and estimated blood loss <250 mls. Two patients achieved durable, satisfactory outcomes with no donor-site morbidity at 24 months. One flap failed after inadvertent division of the dominant perforator, underscoring technical risks in fibrotic planes.

Key lessons include meticulous intramuscular dissection, preservation of a secondary perforator until inset, creation of a generous tunnel to prevent kinking, and early management of venous congestion. In patients unsuitable for abdominal flaps or latissimus dorsi sacrifice, a pedicled TDAP combined with an expandable implant offers a muscle-sparing, single-stage option for delayed reconstruction after implant failure.

## Introduction

First described by Angrigiani et al. in 1995, the thoracodorsal artery perforator (TDAP) flap comprises skin and fat overlying the latissimus dorsi (LD) muscle, supplied by perforators of the thoracodorsal artery, a branch of the subscapular artery [1,2]. By preserving the LD muscle, it reduces donor site morbidity and better preserves shoulder function compared with musculocutaneous flaps [3].

Since Hamdi et al. (2004) first described its use in immediate partial breast reconstruction, the TDAP flap has been widely adopted for reconstruction following oncological resection, burns, and both immediate and implant-based breast reconstructions [4,5]. It has also been used in immediate salvage procedures following failed prosthetic breast reconstructions [6]. Several autologous flaps, including the deep inferior epigastric perforator (DIEP), LD, and muscle-sparing transverse rectus abdominis myocutaneous (ms-TRAM) flaps, are utilized for salvage breast reconstruction [7]. However, these may be unsuitable in low-BMI patients or those lacking abdominal donor tissue, in whom a muscle-sparing TDAP can provide vascularized soft-tissue coverage and, when combined with an implant, restore volume [8,9].

In this report, delayed reconstruction refers specifically to TDAP-based reconstruction performed ≥12 months after failed implant-based reconstruction. This interval allows resolution of infection or inflammation, completion of adjuvant therapy, psychological recovery, and soft-tissue maturation, and reflects NHS scheduling realities for imaging, planning and listing. Unlike studies of immediate/early TDAP or ‘surgical delay’ techniques, this series focuses on a single-stage, hybrid TDAP-plus-expandable-implant approach beyond 12 months after implant failure. We describe three such cases, highlighting patient selection, operative nuances, and practical lessons.

### Pre-operative planning and operative technique

**Figure 1.**
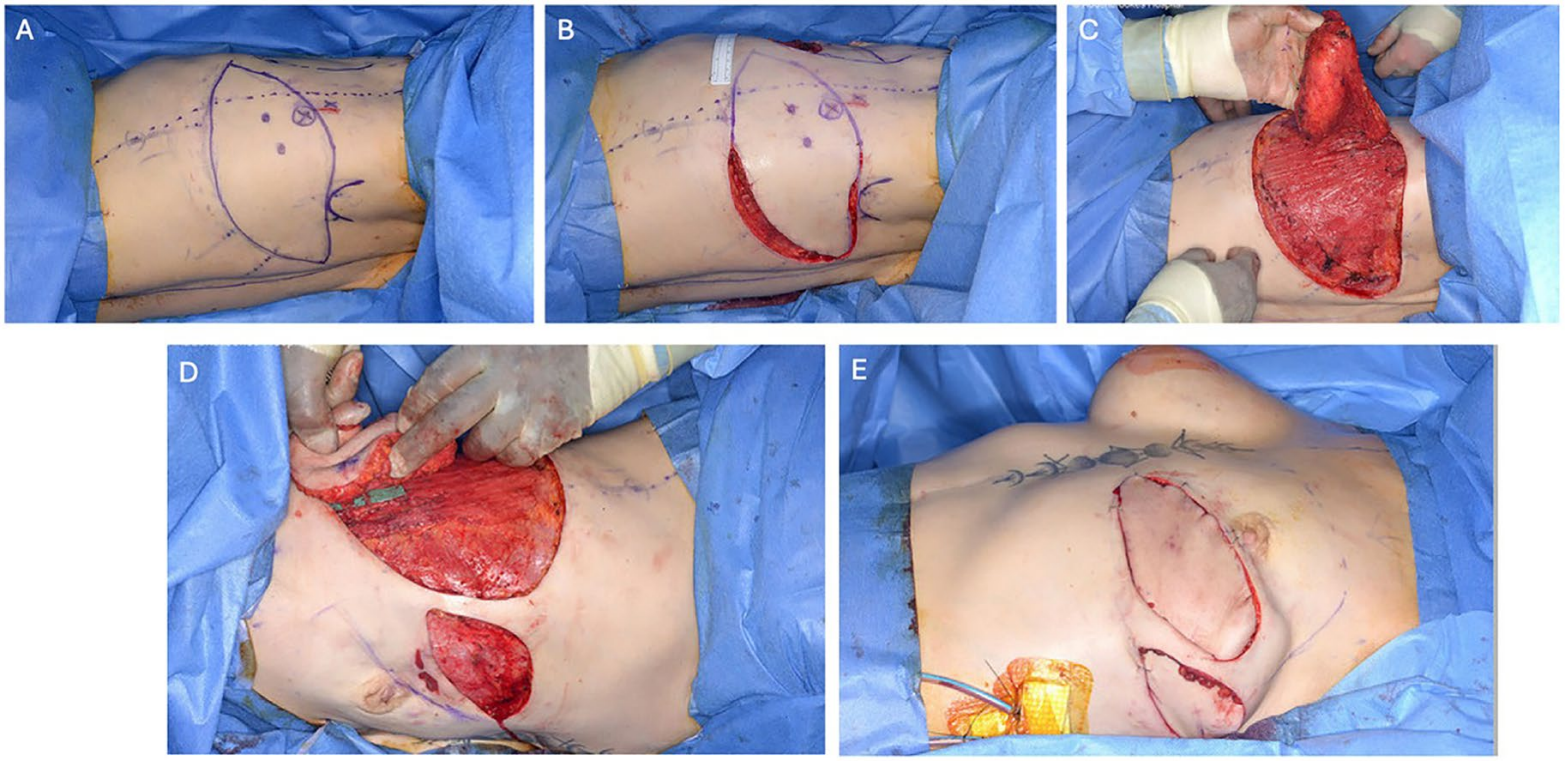
Intraoperative steps of thoracodorsal artery perforator (TDAP) flap harvest and inset. (A) Preoperative Doppler identification and marking of thoracodorsal artery perforators with the patient in the lateral decubitus position. (B) Incision and dissection of the skin paddle down to the muscle fascia. (C) Elevation of the flap from medial to lateral and superior to inferior to expose the dominant perforator. (D) Careful intramuscular dissection of the perforator through the latissimus dorsi muscle with division of side branches. (E) Final flap inset after subpectoral pocket creation, 180° rotation, and tunnelling beneath the split latissimus dorsi muscle.

All patients underwent pre-operative CT angiography to map thoracodorsal perforators. Intraoperatively, perforating vessels of the thoracodorsal artery (TDA) are identified using a handheld Doppler and marked while the patient is in a lateral decubitus position with the elbow flexed at 90 degrees. This helps in designing the skin paddle and selecting the appropriate TDA branch to base the flap on (Figure 1(A)). The flap is harvested with the patient in a lateral position and the shoulder abducted to 90 degrees. The skin paddle is incised, and dissection proceeds down to the muscle fascia (Figure 1(B)). The flap is raised from medial to lateral and from superior to inferior until the dominant perforator is identified (Figure 1(C)). The perforator is meticulously dissected through the LD muscle, with all side branches ligated and divided (Figure 1(D)).

The thoracodorsal nerve is identified and preserved while the muscle is split, and the thoracodorsal pedicle is dissected proximally up to the serratus anterior branch. The anterolateral border of the LD muscle is split and elevated to facilitate flap transposition through the subcutaneous tunnel between the breast pocket and TDAP donor site. In all three patients, the initial implant reconstruction had been performed in a subpectoral pocket with acellular dermal matrix (ADM) because of tenuous overlying skin and the need for pocket control. During delayed reconstruction, we re-used the same subpectoral pocket; the TDAP is rotated 180 degrees clockwise and passed under the split LD muscle (Figure 1(E)). Pedicle length and tunnel width were tailored to ensure tension-free, kink-free transposition of the flap into the existing pocket. Finally, flap perfusion is confirmed using Doppler (indocyanine green [ICG] angiography was not available at our center), and the donor site is closed. Each reconstruction was performed as a single-stage pedicled TDAP flap combined with a subpectoral Becker-35 expandable implant (Table 1).

**Table 1.** Patient characteristics, pre-operative context, and delayed hybrid reconstruction details.

Case	Age	BRCA	BMI	Pre-op RT	Pre-op ADM	Pre-op infection (cause of implant loss)	Side reconstructed	CTA / Doppler / ICG	Flap size (cm)	Pedicle (cm)	OT (min)	EBL (mls)	Pocket	Implant used	Outcome	Follow-up
1	33	BRCA1	23	**Yes**	**Yes**	**Yes**	Left	**CTA: Yes / Doppler: Yes / ICG: No**	**14 × 7**	**14**	**250**	**<200**	**Subpectoral (re-used)**	**Becker-35 expander**	**Uneventful; no donor-site issues**	**24 mo**
2	48	BRCA1	22	**No**	**Yes**	**Yes**	Left	**CTA: Yes / Doppler: Yes / ICG: No**	**20 × 8**	**13**	**220**	**<250**	**Subpectoral (re-used)**	**Becker-35 expander**	**Uneventful; good symmetry**	**24 mo**
3	32	BRCA1	25	**No**	**Yes**	**Yes**	Left	**CTA: Yes / Doppler: Yes / ICG: No**	**16 × 8**	**15**	**270**	**<250**	**Subpectoral (re-used)**	**Becker-35 expander**	**Flap thrombosis; expander retained; small donor-site dehiscence closed**	**12 mo**

Abbreviations: RT, radiotherapy; ADM, acellular dermal matrix (from the index implant reconstruction); CTA, CT angiography; ICG, indocyanine green; OT, operative time; EBL, estimated blood loss.

Notes.

1. Pre-op RT/ADM/infection refer to factors present before the delayed TDAP procedure and explain field hostility (radiation, scar from ADM, infection as the cause of implant loss).

2. All delayed reconstructions were single-stage pedicled TDAP + subpectoral Becker-35 using the existing subpectoral pocket.

3. CTA was performed in all cases; Doppler was used intra-operatively; ICG was not available.

### Case 1

A 33-year-old female with a BRCA1-positive gene mutation underwent bilateral nipple-sparing mastectomies (left therapeutic and right prophylactic) with axillary lymph node clearance in November 2019, followed by immediate bilateral prepectoral breast reconstruction using fixed-volume implants at a neighboring institution. Her bra cup size increased from AA to DD postoperatively. She subsequently received adjuvant radiotherapy and was referred to our plastic surgery service four years later for a second opinion regarding her suboptimal reconstruction. She reported a reduction in breast volume (left more than right), progressive upward displacement, pain and tenderness, particularly affecting her left breast. She was highly dissatisfied with her breast appearance. The patient had a normal body habitus (BMI 23.0) and no viable autologous donor sites.

In November 2023, she underwent bilateral anterior disc capsulectomies with implant exchange from prepectoral fixed volume prostheses to subpectoral expandable implants covered with ADM. The following month, she developed an implant infection refractory to oral antibiotics, necessitating hospital admission for intravenous (IV) antibiotics. Despite treatment, the infection progressed to implant exposure, necessitating surgical exploration and explantation in January 2024.

**Figure 2.**
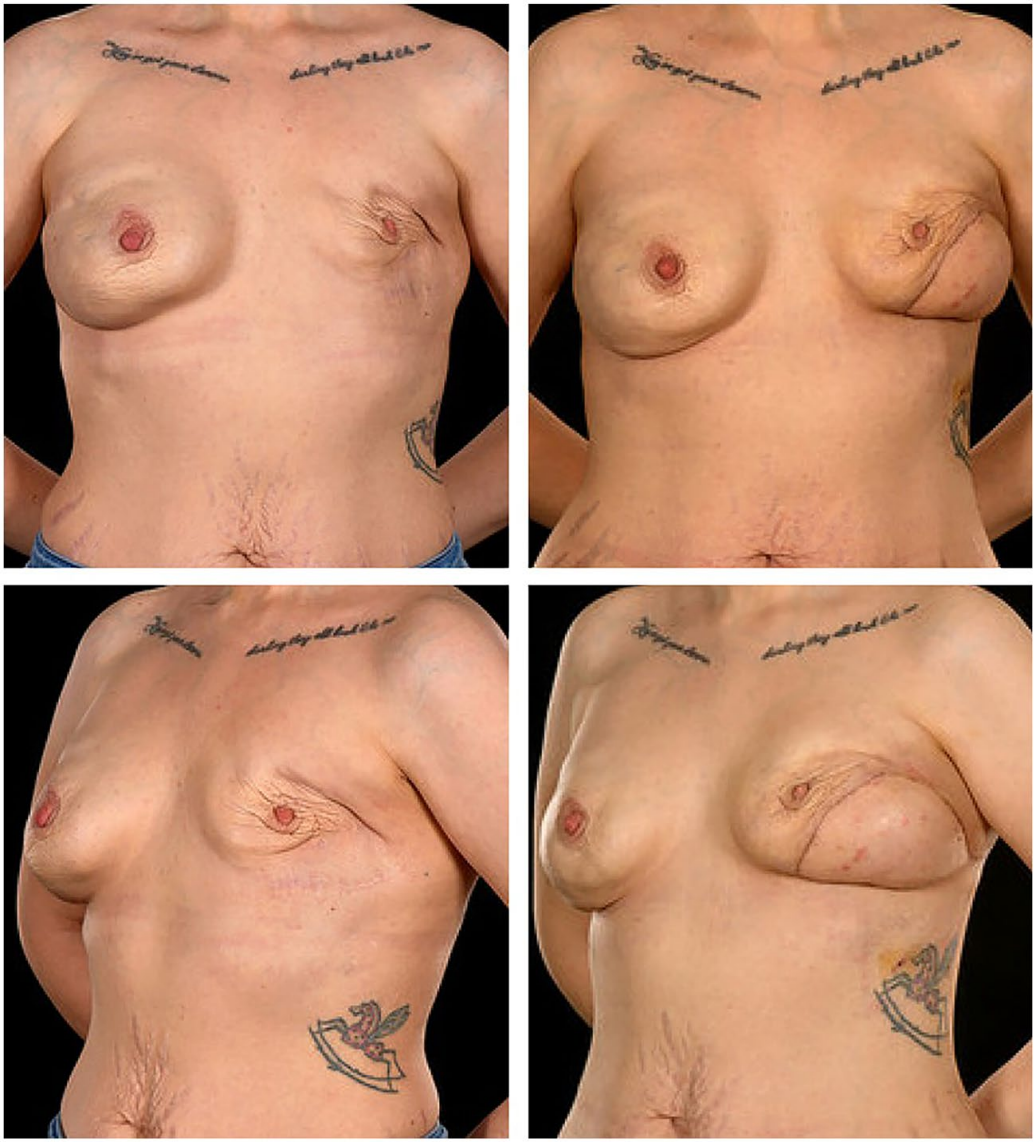
Pre-operative (left) and six-month post-operative (right) anterior and lateral view photographs of Patient 1, following delayed salvage reconstruction of the left breast using a thoracodorsal artery perforator (TDAP) flap combined with a subpectoral Becker-35 expandable implant. The reconstruction resulted in the restoration of breast volume, improved symmetry, and a satisfactory aesthetic outcome.

After 12 months, she underwent salvage of the failed left reconstruction using a 14 × 7 cm pedicled TDAP flap (pedicle 14 cm; operative time 250 mins; blood loss <200 mls) combined with a subpectoral Becker-35 expandable implant. Recovery was uneventful, and 24-month follow-up confirmed a stable result with no donor-site morbidity (Figure 2).

### Case 2

A 48-year-old female (BMI 22) with a BRCA1-positive mutation underwent risk-reducing bilateral nipple-sparing mastectomies and immediate subpectoral breast reconstruction using fixed volume implants and Braxon FAST ADM in March 2024. Within the first four weeks, she developed wound dehiscence resulting in infection and implant exposure. She was admitted for surgical debridement, washout and antibiotics. Despite this, the implant had to be removed and was replaced with an expandable implant. One month later, she developed a peri-implant infection, which ultimately led to implant removal.

**Figure 3.**
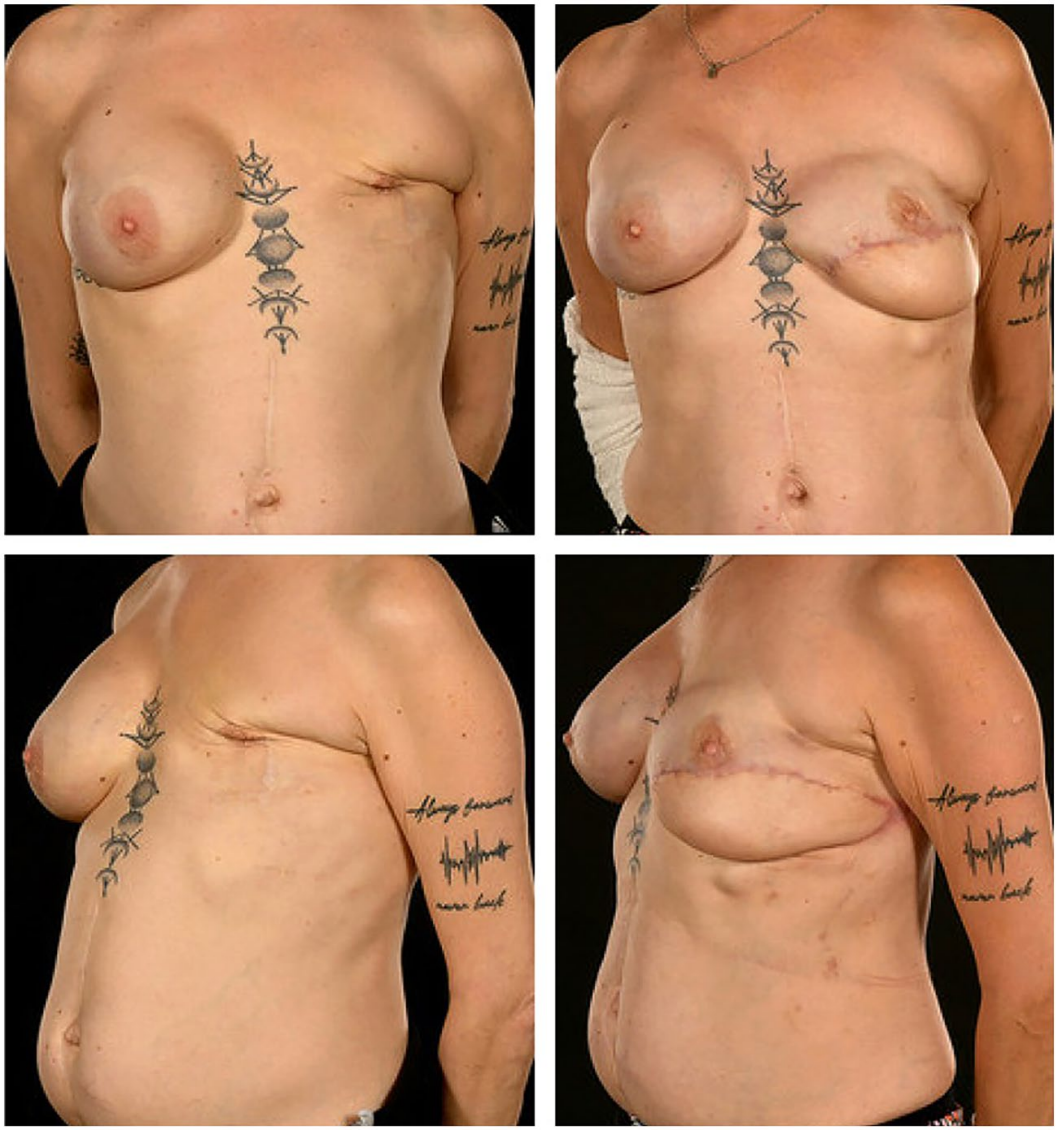
Pre-operative (left) and six-month post-operative (right) anterior and lateral view photographs of patient 2 following delayed salvage of failed left implant-based reconstruction. Reconstruction was performed using a thoracodorsal artery perforator (TDAP) flap combined with a subpectoral Becker-35 expandable implant, achieving an improved aesthetic outcome.

After 12 months, she underwent delayed hybrid reconstruction using a 20 × 8 cm pedicled TDAP flap (pedicle 13 cm; operative time 220 min; blood loss < 250 mls) and a subpectoral Becker-35 expandable implant. Post-operative recovery was uneventful, and the 24-month follow-up demonstrated good symmetry and contour (Figure 3).

### Case 3

A 32-year-old female with a BRCA1-positive mutation and BMI of 22.8 presented with a self-detected breast cancer following completion of neoadjuvant chemotherapy. She underwent a left therapeutic skin-sparing mastectomy (SSM) with sentinel lymph node biopsy (SLNB) and right risk-reducing SSM, followed by immediate bilateral breast reconstruction using subpectoral Mentor Becker-35 expandable implants and Braxon FAST ADM in June 2024. Approximately one month post-operatively, she developed significant wound healing issues and seroma formation on the left side, requiring hospital admission for wound debridement and implant removal.

**Figure 4.**
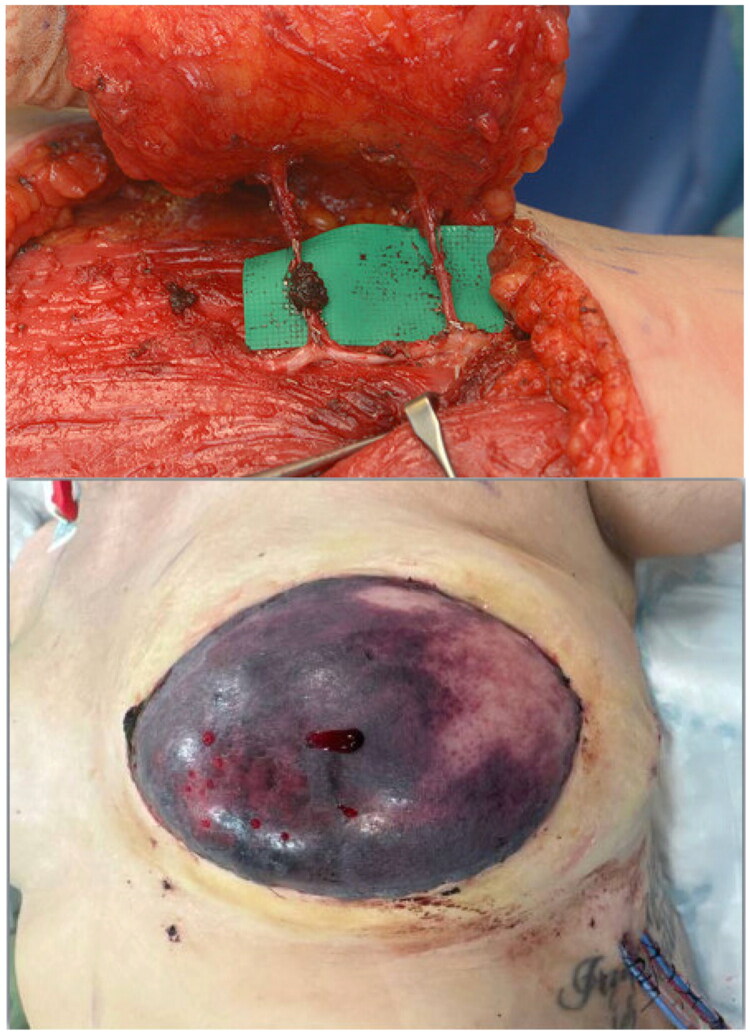
Intraoperative view demonstrating intramuscular dissection of the thoracodorsal artery perforators during TDAP flap harvest in patient 3. The dominant perforator (left) was inadvertently divided while dissecting through the latissimus dorsi muscle. The flap was subsequently raised on a smaller adjacent perforator (right), which initially appeared viable. However, post-operatively, the flap developed venous congestion (below) and ultimately failed due to thrombosis.

12 months later, she underwent delayed hybrid reconstruction of the failed left implant-based reconstruction using a 16 × 8 cm TDAP flap (pedicle 15 cm; operative time 270 min; blood loss < 250 mls) combined with a subpectoral Becker-35 expandable implant. During flap harvest, the dominant perforator was inadvertently divided in dense scar tissue (Figure 4). Consequently, the flap was raised on a smaller perforator, which initially appeared well perfused. Immediately post-operatively, the Doppler signal was difficult to localize; however, an arterial signal was audible. However, following transfer to the ward, the flap developed persistent venous congestion of the inferomedial border. Leech therapy was initiated but provided minimal improvement. She returned to theater the following day, where surgical exploration revealed flap thrombosis and a hematoma beneath the flap. The hematoma was evacuated, and the entire flap was debrided but leaving the expander *in situ*. One month later, the patient developed a 6 cm wound dehiscence at the LD donor site, which was debrided and closed primarily under local anesthetic. The patient recovered well postoperatively.

## Discussion

The TDAP flap is widely used for breast reconstruction, and its various flap-harvesting techniques are well described in the literature [5,9,10]. It provides volume and supple skin for implant coverage, resulting in a natural contour. The flap benefits from a long vascular pedicle, offering greater reach and flexibility. Once a suitable perforator is identified, flap harvest is relatively straightforward [11]. Both the vertical and horizontal branches of the thoracodorsal artery provide perforating branches, allowing skin paddle orientation to be tailored to the reconstructive requirement [12]. In a retrospective study of 126 patients, Homsy et al. demonstrated the effectiveness of the TDAP flap, particularly in patients with limited reconstructive options [13].

In the present series, delayed reconstruction is defined as TDAP-based reconstruction performed ≥ 12 months after failed implant-based reconstruction. This timing allows resolution of infection/inflammation, completion of adjuvant therapy, psychological recovery, and maturation of the recipient soft tissues, while also reflecting NHS scheduling realities. Whilst the TDAP flap has been previously used for immediate or early salvage of failed prosthetic breast reconstructions [6], its use as a single-stage hybrid strategy (TDAP plus expandable implant) beyond 12 months has been rarely described and presents distinct technical challenges due to fibrosis, distorted planes, and potential vascular compromise after prior surgery, infection, or radiotherapy.

Our reconstructive strategy in all three cases was deliberately patient-centred. Each patient wished to avoid multiple staged procedures (e.g. serial fat grafting or delayed implant exchange), and several patient factors limited purely autologous options: low BMI or prior abdominal liposuction, the need to preserve latissimus dorsi function, and poor-quality skin following infection and/or radiotherapy. The pre-existing subpectoral pocket (with ADM from prior surgery) was therefore re-used and reinforced with a pedicled TDAP to provide vascularised soft-tissue cover over the new expandable implant, offering a single-operation solution that aligned with patient preference. Pre-operative CT angiography was used to map perforators and handheld Doppler to confirm them intra-operatively; ICG angiography was unavailable in our unit.

### Comparison with alternative flaps

DIEP/ms-TRAM flaps provide excellent volume but require adequate abdominal tissue and longer operative times and were unsuitable in these low-BMI/abdominally compromised patients. The LD-plus-implant option remains reliable but sacrifices muscle and risks shoulder-function morbidity. Local perforator flaps such as LICAP/AICAP/MICAP have an established role, particularly for lateral defects, but their shorter pedicles can limit central/medial reach in scarred fields [4]. In our patients, where the principal problem was central soft-tissue deficiency over an implant in a hostile bed, the TDAP’s longer pedicle and versatile skin-paddle design allowed stable central coverage and pocket protection in a single stage. Compared with serial fat grafting, the hybrid TDAP-plus-implant approach avoids multiple admissions/anesthetics and provide immediate projection and contour.

### Lessons from the failed case (risk-mitigation checklist and algorithm)

In Case 3, the dominant perforator was inadvertently divided during intramuscular dissection in fibrotic LD, and venous congestion developed despite an initial arterial Doppler signal. This experience has refined our protocol: (i) pre-operative CTA mapping with intra-operative Doppler confirmation; (ii) optical magnification and minimal thermal injury during intramuscular dissection; (iii) preservation of a secondary perforator until final inset confirms robust perfusion; (iv) ensuring adequate pedicle length and a generous, tension-free tunnel over the implant to prevent kinking or compression; and (v) a structured venous-congestion algorithm - release constricting sutures/dressings, consider partial ‘unroofing’ or turndown of the flap, commence leech therapy for superficial congestion, and maintain a low threshold for early re-exploration to exclude haematoma or pedicle torsion. ICG angiography may assist decision-making in scarred fields where available.

### Technical and outcome summary

Across the series, flap dimensions were 14–20 × 7–8 cm with pedicles 13–15 cm; operative time was 220–270 min and blood loss < 250 mls. Follow-up was 24 months for Cases 1–2 and 12 months for Case 3. At up to 24 months’ follow-up, Cases 1–2 demonstrated stable aesthetic results with no donor-site morbidity or implant exposure, while Case 3 resulted in flap loss with the expander retained (grade IIIb Clavien-Dindo). These outcomes are consistent with larger series reporting TDAP safety and versatility in complex breast reconstruction [13].

### Broader perspective

We acknowledge that TDAP flaps are technically demanding, offer limited intrinsic volume, and, when combined with an implant, carry a recognised risk of venous congestion. However, in experienced hands and with the risk-mitigation steps outlined above, these risks are manageable. For carefully selected patients who lack suitable abdominal tissue, wish to avoid LD sacrifice, and prefer to minimise staged procedures, a single-stage TDAP-plus-implant reconstruction provides a pragmatic, muscle-sparing option in the delayed setting.

## Conclusion

The pedicled TDAP flap, combined with an expandable subpectoral implant, represents a feasible single-stage hybrid option for delayed (≥ 12 months) reconstruction after failed implant-based breast surgery. In scarred fields, careful CTA-guided planning, meticulous intramuscular perforator dissection, and a structured approach to venous-congestion management can deliver stable coverage and aesthetic restoration while avoiding the morbidity of abdominal harvest or latissimus dorsi sacrifice.

## References

[1] Thoracodorsal Artery Perforator Flap (TAP Flap). https://www.microsurgeon.org/tapflap. (accessed 2025-02-27).

[2] Angrigiani C, Grilli D, Siebert J. Latissimus dorsi musculocutaneous flap without muscle. Plast Reconstr Surg. 1995;96(7):1608–1614. doi: 10.1097/00006534-199512000-00014. 7480280

[3] Abdelrahman EM, Nawar AM, Balbaa MA, et al. Oncoplastic volume replacement for breast cancer: latissimus dorsi flap versus thoracodorsal artery perforator flap. Plast Reconstr Surg Glob Open. 2019;7(10):e2476. doi: 10.1097/GOX.0000000000002476. 31772899 PMC6846317

[4] Hamdi M, Van Landuyt K, Monstrey S, et al. Pedicled perforator flaps in breast reconstruction: a new concept. Br J Plast Surg. 2004;57(6):531–539. doi: 10.1016/j.bjps.2004.04.015. 15308400

[5] Mangialardi ML, Baldelli I, Salgarello M, et al. Thoracodorsal artery perforator flap in partial breast reconstruction: a systematic review. Plast Reconstr Surg Glob Open. 2020;8(10):e3104. doi: 10.1097/GOX.0000000000003104. 33173666 PMC7647658

[6] Nizamoglu M, Hardwick S, Coulson S, et al. The use of the thoracodorsal artery perforator flap in both autologous and implant based breast reconstruction salvage surgery. Clin Surg. 2020;5:3006.

[7] Coriddi M, Shenaq D, Kenworthy E, et al. Autologous breast reconstruction after failed implant based reconstruction; evaluation of surgical and patient reported outcomes and quality of life. Plast Reconstr Surg. 2019;143(2):373–379. doi: 10.1097/PRS.0000000000005197. 30688876 PMC6352728

[8] Themes, U. F. O. Partial Breast Reconstruction With Flaps. Plastic Surgery Key. https://plasticsurgerykey.com/partial-breast-reconstruction-with-flaps/. (accessed 2025-02-27).

[9] Angrigiani C, Rancati A, Escudero E, et al. Extended thoracodorsal artery perforator flap for breast reconstruction. Gland Surg. 2015;4(6):519–527. doi: 10.3978/j.issn.2227-684X.2015.04.20. 26645006 PMC4647016

[10] Thomsen JB, Rindom MB, Rancati A, et al. Thoracodorsal artery flaps for breast reconstruction–the variants and its approach. Arch Plast Surg. 2021;48(1):15–25. doi: 10.5999/aps.2020.01410. 33503740 PMC7861974

[11] Jain L, Kumta SM, Purohit SK, et al. Thoracodorsal artery perforator flap: indeed a versatile flap. Indian J Plast Surg. 2015;48(2):153–158. doi: 10.4103/0970-0358.163051. 26424978 PMC4564498

[12] Heitmann C, Guerra A, Metzinger SW, et al. The thoracodorsal artery perforator flap: anatomic basis and clinical application. Ann Plast Surg. 2003;51(1):23–29. doi: 10.1097/01.SAP.0000054189.14799.F3. 12838121

[13] Homsy C, Theunissen T, Sadeghi A. The thoracodorsal artery perforator flap: a powerful tool in breast reconstruction. Plast Reconstr Surg. 2022;150(4):755–761. doi: 10.1097/PRS.0000000000009576. 35862116

